# Supplementary material for: The Impact of Omega-3 Supplements on Non-Surgical Periodontal Therapy: A Systematic Review
Source: Nutrients. 2022 Apr 28;14(9):1838. doi: 10.3390/nu14091838 (PMC9105356; doi:10.3390/nu14091838)
Supplement: Supplementary file 1 [file nutrients-14-01838-s001.zip › nutrients-1628148-supplementary.pdf]

Supplementary Table S1: Data about dental plaque and oral hygiene instructions (OHI) and reinforcement (OHR) along over the study period.

Authors, year	Groups	PI baseline	PI final	Mean PI reduction	P-value intra-group	P-value inter-group	OHI	OHR
Deore et al., 2014 [41]	T	2.45 ±030	1.57 ±0.26	0.88	p<0.05	p>0.05	Yes	Yes
	C	2.37 ±0.39	1. 55 ±0.29	0.82	p<0.05		Yes	No
Suramya et al., 2014 [39]	T	2.31 ±0.42	1.10 ±0.11	1.21	p<0.05	p>0.05	No	No
	C	2.27 ±0.28	1.26 ±0.21	1.01	p<0.05		No	No
Keskiner et al., 2017 [43]	T*	1.90(1.12-2.62)	1.16(0.56-1;66)	0.74	p<0.05	p>0.05	Yes	No
	C*	1.88(1.66-2.27)	1.07(0.31-1.43)	0.81	p<0.05		Yes	No
El-Sharkawy and Elmeadawy., 2017 [42]	T	2.43 ±0.36	0.79 ±0.35	1.64	p<0.001	p>0.001	Yes	No
	C	2.38 ±0.86	0.86 ±0.47	1.52	p<0.001		Yes	No
Elgendy and Kazem, 2018 [40]	T	1.69 ± 0.57	0.36 ±0.19	1.33	p<0.05	p>0.05	Yes	Yes
	C	1.73 ±0.55	0.42 ±0.20	1.31	p<0.05		Yes	Yes
Rampally et al., 2019 [38]	T	NR	NR	NA	NA	NA	No	No
	C	NR	NR	NA	NA		No	No
Shalaby and Morsy, 2019 [44]	T	35 ±21	17 ±9	18 ±19	p<0.001	p>0.001	No	No
	C	49 ±19	34 ±16	15 ±23	p<0.001		No	No
Stando et al., 2020 [45]	T	2.13 ±0.57	0.51 ±0.25	1.62	p<0.05	p>0.05	Yes	Yes

T: test group; C: control group; NA: not applicable; NI: not informed; NR: not recorded; NS: OHI: oral hygiene instructions; OHR: oral hygiene reinforcement; PI: plaque index; *Median and Percentiles 25-75
